# A Behavioral Mechanism of How Increases in Leg Strength Improve Old Adults’ Gait Speed

**DOI:** 10.1371/journal.pone.0110350

**Published:** 2014-10-13

**Authors:** Azusa Uematsu, Kazushi Tsuchiya, Norio Kadono, Hirofumi Kobayashi, Takamasa Kaetsu, Tibor Hortobágyi, Shuji Suzuki

**Affiliations:** 1 Faculty of Human Sciences, Waseda University, Tokorozawa, Japan; 2 Graduate School of Human Sciences, Waseda University, Tokorozawa, Japan; 3 Advanced Research Center for Human Sciences, Waseda University, Tokorozawa, Japan; 4 Graduate School of Arts and Sciences, The University of Tokyo, Meguro-ku, Japan; 5 School of Human Sciences, Waseda University, Tokorozawa, Japan; 6 University Medical Center Groningen, University of Groningen, Groningen, The Netherlands; 7 Department of Sport, Exercise and Rehabilitation, Northumbria University, Newcastle-upon-Tyne, United Kingdom; West Virginia University School of Medicine, United States of America

## Abstract

We examined a behavioral mechanism of how increases in leg strength improve healthy old adults’ gait speed. Leg press strength training improved maximal leg press load 40% (p = 0.001) and isometric strength in 5 group of leg muscles 32% (p = 0.001) in a randomly allocated intervention group of healthy old adults (age 74, n = 15) but not in no-exercise control group (age 74, n = 8). Gait speed increased similarly in the training (9.9%) and control (8.6%) groups (time main effect, p = 0.001). However, in the training group only, in line with the concept of biomechanical plasticity of aging gait, hip extensors and ankle plantarflexors became the only significant predictors of self-selected and maximal gait speed. The study provides the first behavioral evidence regarding a mechanism of how increases in leg strength improve healthy old adults’ gait speed.

## Introduction

The decline in self-selected habitual gait speed can reach 16% per decade starting at age 60 [1]–[5]. A decline in gait speed is normally associated with deteriorating health. Consequently, self-selected habitual gait speed measured on a level surface in middle ages predicts many clinical conditions later in life, including daily function, late-life mobility, independence, falls, fear of falls, fractures, mental health, cognitive function, adverse clinical events, hospitalization, institutionalization, and survival [2], [6]–[15]. A gait speed of ∼0.6 m/s has been identified as a threshold for frailty and represents an additional risk factor for functional decline in old adults [2]. Therefore, maintaining gait speed over this threshold or even over 0.8 m/s, as suggested in some studies, is clinically important because slow gait can potentially lead to motor passivity: as the time needed to cross the street or reach a destination becomes longer, old adults become less motivated to ambulate [16].

Leg strength and power can help maintain gait speed in old age. Some studies reported a significant association between gait speed and old adults’ ability to produce force during a maximal voluntary contraction (MVC). For example, plantarflexor [4] and knee extensor MVC forces [17], [18], and leg power [3], [19]–[21] are associated with gait speed. In other studies, however, hip and knee extensor strengths did not correlate with gait speed [22]. Novel compared with a simple type of exercise interventions tend to increase gait speed somewhat more reliably [8]. However, a careful analysis in recent review revealed a statistically significant but weak association between the training-induced increases in leg muscle strength and gait speed (r^2^ = 0.21, p = 0.018) [23]. Furthermore, the coefficient of determinations between increases in plantarflexor MVC and gait speed in 6 studies and between the increases in leg power and gait speed in 8 studies, respectively, was r^2^ = 0.16 (p = 0.421), and r^2^ = 0.00 (p = 0.996) [23]. Therefore, the mechanism of how strength and power training increases gait speed remains unclear.

A critical element that these studies have not yet addressed is how, if at all, does a strength intervention affect the relationship between leg strength and gait speed. More specifically, it is unknown as to how the increase of an individual leg muscle group’s strength contributes to gait speed after the intervention and if muscles that improve the most also predict the increases in gait speed most accurately. Because the ankle plantarflexors contribute over 70% to the total leg mechanical output in the propulsion phase of gait [23], even small increases in ankle strength could play a significant role in increasing gait speed. Yet, old compared with young adults use their hip extensors more and ankle planter flexors less during gait [24]. This is the concept of biomechanical plasticity of the aging gait [23], [24] that suggests a shift in function to the proximal hip from distal ankle extensors, implying that any increase in hip function could hypothetically be also a factor in improving gait speed. Taken together, the purposes of the present study were: 1) to determine the effects of lower extremity strength training on maximal strength of 5 leg muscle groups including hip abductor, hip adductors, hip extensors, knee extensors, and ankle plantarflexors; 2) to examine the relationship between leg strength of each muscle group and gait speed before and after the intervention; and 3) to test the hypothesis that, according to the concept of biomechanical plasticity of gait, hip and ankle functions become significant predictors of gait speed in healthy old adults. If these expectations were correct, the results would provide the first behavioral evidence regarding the mechanism of how increase in leg strength actually improves gait speed in old adults.

## Methods

### Ethics statement

The Human Ethics Committee at Waseda University approved the study protocol. Before the start of the study, each participant read and signed a written informed consent. All procedures were conducted according to the Declaration of Helsinki.

### Subjects and design

Twenty-five 70–81-year-old healthy community-dwelling volunteers (12 males, 13 females) participated in this study. All participants were healthy enough to visit the laboratory on their own by car, bicycle, or on foot.

The study had a 2-group pre-test and post-test design. We assigned participants randomly to control (3 males and 5 females; age = 74.3±3.9 years; height = 156.3±10.6 cm; weight = 54.3±10.7 kg) and exercise-training groups (9 males and 8 females). One female participant withdrew from the study due to knee joint pain caused by an accident. Another female participant dropped out of the study after the 1st training session due to time conflicts. Thus, 9 males and 6 females (age = 74.3±3.4 years; height = 158.8±6.8 cm; weight = 57.7±7.3 kg) completed all the training and testing sessions. We tested all participants for walking ability, physical performance, and isometric strengths of 5 muscle groups at baseline and after 8 weeks of leg press training or no training.

### Testing protocol

Participants completed the short physical performance battery (SPPB) and maximal isometric strength testing of five muscle groups against a hand-held dynamometer on the initial day of the study. The 1-repetition maximum (1RM) leg strength was tested 2 or 3 days later on a second testing day in the training group.

#### Short physical performance battery (SPPB)

The SPPB is valid and reliable functional tests of old adults’ balance, walking speed, and leg strength [25]. Briefly, SPPB quantifies standing balance, gait speed over 4 m level surface, and leg strength through 5, timed chair rises. Subjects were allowed to practice as needed so that they could become familiar with these tests. Experimenter recorded the time required to perform each test using a stopwatch (S123-4000, SEIKO).

#### Walking speed

We measured participants’ self-selected walking speed on flat surface over 4 m and maximal walking speed on flat surface over 7 m. The 4-m self-selected walking speed was determined as a part of the SPPB test. The instruction was as follows: “Walk at your usual speed, just as if you were walking down the street to go to the store.” For the maximal walking speed, participants stood behind a line and walked past a target line set at 7 m. The instruction was: “Walk as fast as you safely can as if you tried to catch a bus but do not run”. Subjects practiced the maximal walking speed test 1–2 times before the data collection. Walking speed was recorded to the 10th of a second accuracy with the stopwatch.

#### Leg strength

We measured maximal voluntary isometric strength of the right leg during hip extension, abduction, and adduction and during knee extension and ankle plantarflexion using a hand-held dynamometer (MT-100, SAKAI Medical Company, Japan). We adopted a highly standardized testing protocol validated previously (Intraclass correlation coefficients >0.90) [26], [27]. We performed all of the measurements with a calibrated load-cell sensitive to pull forces up to 980 N (MT-110, SAKAI Medical Company, Japan), placed in series within a 2.5-cm wide non-stretch plastic strap. For hip extension, subjects lay prone across an examination table with the left leg straight and the right knee flexed 90° and positioned 10–15 cm past the table edge. The top end of the strap loop was just superior to the popliteal fossa and the other end was anchored tautly to the floor. Subjects performed maximal hip extension by pressing upwards into the strap. For hip abduction, subjects lay supine with both legs straight on the examination table 30° apart and the strap looping around both knees. Subject performed maximal hip abduction by pressing the straight right leg outward into the strap. Subjects assumed the same position on the table for hip adduction, but the strap was looped around the subject’s right knee and around the left thigh of the experimenter, standing next to the examination table on the right side of the subject. For knee extension, subjects sat in an armless chair and flexed the knee 90°. One end of the strap loop was placed on the shank just above the right lateral malleolus and the other end around the right rear leg of the chair. The foot sole was about 1 cm above the floor. For ankle plantarflexion, subjects assumed the same body position as for hip extension. One end of the strap loop was around the distal end of the metatarsals and the other end around the table leg. The ankle was in anatomical position. For all tests, subjects were allowed to grasp the table or chair edge. Participants first practiced each test at 40–50% of maximal effort then performed 1–2 trials at 70–80% of maximal effort. Subjects performed 2 maximal effort trials for 5 seconds each with strong verbal encouragement. There was 30 s of rest between each strength test. One researcher administered all of the strength tests in a randomized order between subjects. The higher of the 2 scores was used in the analysis.

#### 1RM leg strength

On a separate day, we measured leg strength during a bilateral 1RM seated leg press on a weight-stacked leg press machine (maximum load: 218 kg, Nautilus), used also in the exercise training intervention. Subjects started the leg press movement with the hip and knee joints flexed 90° and completed the movement with the hip and knees extended and plantarflexed. Subjects were instructed to complete the press movement in 1 continuous, smooth, and brisk motion and then return to the starting position slowly and smoothly. After a thorough familiarization with the test, subjects performed several trials at 30–40% and then 2–3 trials at 70–80% of their anticipated maximal load. The load, based on preliminary work, was then increased in about 9 kg steps until the 1RM load. Subjects performed no more than 4 steps to reach 1RM and had no difficulty in executing the leg press testing. There was 60 s of rest between each trial. To minimize the potential effects of 1RM testing on mobility, only the training group performed the 1RM testing.

### Training protocol

We chose leg press as an exercise stimulus because preliminary work revealed that the electromyographic activation of the hip, knee, and ankle extensors during leg press was ∼80% of activation measured during single-joint exercise and because these muscles are also involved in the stance phase of gait. For example, compared with a single joint maximal voluntary isometric contraction, during a 1RM leg press the activation of the hip extensor biceps femoris, the knee extensor vastus lateralis, and the ankle plantarflexor gastrconemius medialis was, respectively, 87% (±16), 92% (±22), and 78% (±19) (n = 6 young adults, age 21.2).

The training program consisted of 16 sessions administered over 8 weeks. Each session was about 30 minutes long and started with a standard 5–10 minutes of warm-up that included: rhythmical bouncing and bending exercise, stretching of hip, knee, and ankle muscles, side-to-side torso flexion, and arm rotations. The training load was then set at 30–40% of 1RM and participants performed 3 bouts of 10 repetitions. Subjects remained seated in the leg press machine during the 2-minutes-long inter-bout rest period. Subjects were instructed to perform each repetition rapidly and forcefully. The training load was adjusted throughout the training program. We selected a light load, higher movement velocity to increase the specificity of the training stimulus to gait speed, as suggested previously [28]–[30]. The same staff person supervised each training session. After the training, there was a 5-minute-long cool down period and subjects performed stretching. Members of the control group continued their habitual activities.

#### Statistical analyses

The data are reported as mean ± SD. The main analysis was a Group (intervention, control) by Time (pre, post) analysis of variance repeated-measures on Time. In case of a significant interaction, a Bonferroni’s post-hoc contrast was used to determine the means that were different. We computed a Pearson’s product correlation coefficient between gait speed and expressions of leg strength. We used a step-wise, multiple-regression analysis to directly test the hypothesis as to which muscle group predicted gait speed after the intervention. We also compared the simple linear regression coefficients before and after training using a small sample t-test. Statistical significance was set for all analysis at p<0.05.

## Results

### Training stimulus

In each of the 8 weeks, the mass lifted by the subjects during the leg press averaged 44 kg (or 39% of the pre-test 1RM), 53 kg (47%), 62 kg (55%), 71 kg (63%), 79 kg (70%), 86 kg (76%), 89 kg (79%), and 90 kg (79%), respectively. The average training load was a moderate 59% relative to pre-test 1RM. Because we instructed the participants to move the loads rapidly, the combination of training volume, movement speed, and the number repetitions – as planned - qualified the training stimulus as power training.

### Changes in SPPB and 1RM

There was no group by time interaction for the SPPB score (p = 0.162) and the groups were also similar at baseline (p = 0.064). The time main effect revealed 7.5% or 0.8 units of improvement (p = 0.002), caused by the 11.1% in the control compared with the 4.2% change in training group. Leg press training improved leg press 1RM 40.4% (±26.3) to 155.9 (±46.2) from 113.0 kg (±35.6) at baseline (p<0.001).

### Changes in gait speed and muscle strength

Table 1 shows that there was no group by time interaction for the 4-m self-selected (p = 0.340) and 7-m maximal (p = 0.560) gait speed, respectively. The time main effect revealed 11.2% or 0.12 m/s improvements in self-selected (p = 0.001) and 7.2% or 0.11 m/s increases in maximal gait speed (p = 0.001). The 2 groups’ gait speeds were similar at baseline (control vs. training group: self-selected: 1.10 vs. 1.05 m/s; maximal: 1.43 vs. 1.56 m/s, both p>0.05).

**Table 1:** Changes in the dependent variables.

Parameter	Interaction/maineffect	Group	Pre	Post
Gait speed (m/s)				
Self-selected	Time	CG	1.10±0.18	1.19±0.14
		EG	1.05±0.15	1.19±0.16
Maximal	Time	CG	1.43±0.16	1.56±0.13
		EG	1.56±0.21	1.66±0.24
Maximal strength (kg)	Group×Time			
Plantarflexion		CG	8.26±4.37	8.85±5.13
		EG	8.13±5.76	12.87±4.83
Knee extension		CG	22.69±6.65	21.76±7.69
		EG	27.97±13.22	29.13±14.69
Hip extension		CG	11.39±6.70	14.20±8.30
		EG	13.57±11.08	22.69±14.00
Hip abduction		CG	16.26±7.04	17.13±4.57
		EG	18.20±5.41	20.46±6.03
Hip adduction		CG	14.33±5.32	14.11±4.89
		EG	16.13±5.26	18.01±6.48

Values are mean ± SD. CG, control group. EG, exercise group. Group×Time, group by time interaction (p = 0.002). Time, time main effect (all p = 0.001).

Table 1 also shows the changes in maximal voluntary strength measured by manual muscle testing in 5 muscle groups. There was no group by muscle group by time interaction (p = 0.394). The group by time interaction (p = 0.002) revealed that the 32.0% or 4.0 kg average improvement in the 5 muscle groups combined was greater than the 6.3% or 0.6 kg gain in the control group (p = 0.002). When each muscle group’s contribution to the summed strength score was computed in the training group before and after the intervention, there was a muscle group by time interaction (p = 0.004). Table 2 shows that the plantarflexors (3.7%) and hip extensors (6.8%) increased and the knee extensors decreased (5.9%) the contribution to the changes in total strength (all p<0.001). The hip abductors and adductors decreased ∼2% the contributions to the total strength change (both p = 0.060).

**Table 2:** Contribution by each of the 5 muscle groups to the summed strength score in the training group (n = 15).

Muscle group	Pre	Post	Significance
Plantarflexors	8.99±3.42	12.73±3.42	**
Knee extensors	33.14±5.41	27.23±3.81	**
Hip extensors	14.54±5.47	21.34±6.48	**
Hip abductors	23.01±5.04	20.84±5.41	
Hip adductors	20.31±4.83	17.87±2.97	

Values are percent of the summed strength score for each muscle (mean ± SD). Time by Muscle group interaction (p = 0.004).

**, significant change between pre and post (p<0.01).

### Correlations analyses

Table 3 shows the correlation coefficients between walking speed and leg strength in the exercise group. Before the intervention, the correlations (range: r = 0.00 to 0.55) were low and except for 1 (hip abduction) of 10 correlation coefficients, not significant (p>0.05). After the intervention, 8 of 10 correlations were statistically significant, ranging from r = 0.53 (p = 0.040) to r = 0.75 (p = 0.001). In 4 of 5 muscles the coefficients were somewhat higher for the maximal (r = 0.60) compared with the self-selected gait speed (r = 0.53). The increase in the correlation was similar between self-selected gait velocity and leg strength (r = 0.32 increase in correlation) and maximal walking speed and leg strength (r = 0.27 increase in correlation).

**Table 3:** Pearson product moment correlation coefficients between maximal leg strength and gait speed before and after exercise intervention (n = 15).

Maximal strength	Gait speed	Pre		Post	
		r	p	r	p
Plantarflexion	Self-selected	−0.01	0.98	0.50	0.06
	Maximal	0.02	0.54	0.75	0.00
Knee extension	Self-selected	0.34	0.21	0.56	0.04
	Maximal	0.51	0.06	0.56	0.03
Hip extension	Self-selected	0.06	0.84	0.58	0.02
	Maximal	0.16	0.56	0.54	0.04
Hip abduction	Self-selected	0.36	0.19	0.56	0.00
	Maximal	0.55	0.03	0.61	0.02
Hip adduction	Self-selected	0.29	0.29	0.47	0.08
	Maximal	0.42	0.12	0.53	0.04

p, 2-tailed probability values for the correlation coefficient.

**Figure 1:**
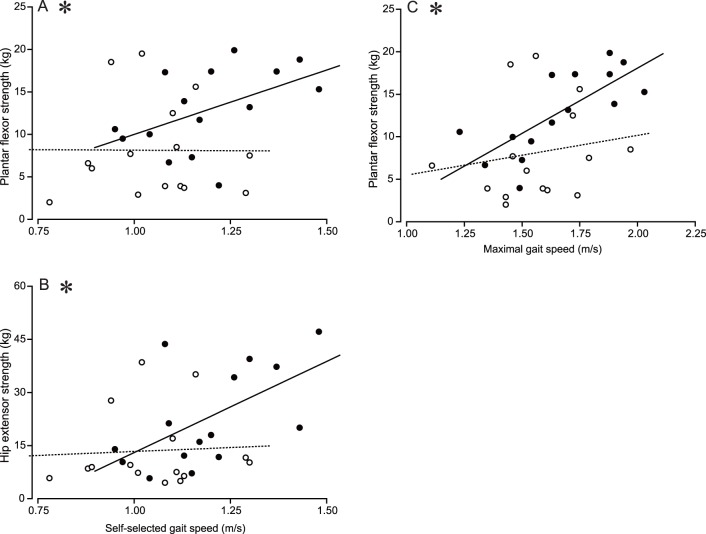
Linear regression of self-selected and maximal gait speed on leg strength before (open symbols, dashed regression lines) and after 16 sessions of leg press training (n = 15). Panel A: before, y = 8.38–0.24x, after, y = 8.84+15.16x; Panel B: before y = 8.89+4.44x, after, y = −35.80+49.91x; Panel C: before, y = 0.84+4.66x, after, y = −12.92+15.50x. The * denotes a significant (p<0.05) difference in the slopes of the regression coefficient before and after training determined by small sample t-test.

At baseline, a forward step-wise, multiple-regression selected hip abductor strength as significant predictor of maximal gait speed (adjusted R^2^ = 0.25, p = 0.034). After the intervention, hip extensor strength became the predictor of self-selected walking speed (adjusted R^2^ = 0.29, p = 0.024) and plantar flexor strength predicted maximal gait speed (adjusted R^2^ = 0.53, p = 0.001). Finally, the regression coefficient became significant for the relationship between self-selected walking speed and plantarflexor and hip extensor strength, respectively (Figure 1, panel A and B) and between maximal gait speed and plantarflexor strength (Figure 1, panel C) (all p<0.05).

## Discussion

The main finding of the present study was that 40.4% increase in leg press 1RM training load and 32.0% increase in maximal isometric strength in 5 leg muscles did not improve gait speed more (9.9%) than what the changes were in the control group (8.6%). However, after the exercise intervention there was a modification in how 5 measures of leg strength contributed to gait speed with hip extensors and ankle plantarflexors becoming the only significant predictors of self-selected and maximal gait speed. We discuss these findings within the framework of biomechanical plasticity of the aging gait.

Leg strength interventions normally improve gait speed [8], [23]. Based on 20 studies, the 22% increase in leg strength was coupled with an 11.7% increase in self-selected gait speed (1.25 to 1.38 m/s) in 815 healthy old adults age 72 [23]. In several of the 20 studies the changes in gait speed were not significant [31] reaching only 5 to 7% [32]–[34] with some studies reporting actually up to 7% decrease instead of an increase in gait speed [35]–[37]. Therefore, our results are not an unusual finding that a strength or power intervention resulted in modest increases in gait speed. Adjusting for height removed the large difference in gait speed between samples consisting of predominantly Caucasian subjects in the review (1.25 m/s) and studies that enrolled Japanese participants only (the present study and [38]) (1.05 m/s) (0.77 vs. 0.76 speed/height ratio), suggesting that the height-adjusted baseline gait speed was similar in our sample and those published previously. Similarly to the 11.7% increase in gait speed in the 20 studies, our subjects increased self-selected gait speed 13.3%. The 8.2% increase in the control group occurred despite rigorously standardized instructions and a measurement environment that was, to smallest details, kept identical throughout the study, suggesting a learning effect due to the repeat testing. The same phenomenon was also observed previously [39], [40]. Therefore, we assume that gait speed can also increase in control subjects as a result of learning.

According to exercise prescription guidelines and recent recommendations, our participants performed power training by moving light-medium loads rapidly [21], [41]. It has been suggested that power vs. strength training may be more suitable to increase gait speed [19], [21], [42]–[44]. While our and other types of power training was successful and improved leg power and strength 34.7% based on data from 8 studies in 150 subjects age 73, a 13.1% increase in self-selected gait speed to 1.38 from 1.25 m/s after those interventions was similar to the 11.7% increase produced by strength training [23]. Thus, the data from the literature suggest that strength and power interventions can improve self-selected gait speed and these changes are often similar after the 2 types of intervention. At least under the current experimental conditions, in healthy but sedentary seniors the exercise adaptations could include a substantial element of learning induced by the testing protocol. This conclusion is further confirmed by the 9.0 and 6.4% increase in maximal gait speed in the control and intervention groups, respectively (time main effect, p = 0.001), a variable that is less often reported in intervention studies [29], [30], [40]. In particular, considering the ∼1.0 m/s self-selected gait speed at baseline, which is well above the 0.8 m/s designated as a marker for median life expectancy [10] and the 0.6 m/s as a threshold for frailty [2], it is likely that the subjects’ mobility level at baseline in our and these previous studies [29], [30], [40] was too high and a ceiling effect blunted the gait responses to the training intervention. Another reason could be that the laboratory environment was restrictive and instructions were inadequate stimuli for participants to motivate themselves and walk faster [45].

The disproportionality between the large increase in leg strength and the clinically small increase in (maximal) gait speed suggests the possibility that an increase in leg strength in high-mobility old adults does not correlate with an increase in gait speed in a simple manner. Previous studies reported that leg muscle strength declines gradually with age [46] but the usual gait speed can decrease rapidly over age 80 [47]. Participants in the present study had such levels of leg muscle strength at baseline that affected functional abilities to only a small extent. Although the intervention was successful in increasing leg strength, the increases in gait speed in the intervention group were much smaller because the functional level in gait was higher compared with the age-related level of relative strength loss. A logical next step for future studies is to examine neuromuscular adaptations to strength and power interventions and determine if such adaptations explain more of the variation in gait speed in functionally less capable old adults than the subjects in the present study.

Leg press has been often a choice of exercise to target the lower extremity extensor mechanism in an effort to improve mobility, including gait speed in old age [28], [29], [39], [40]. A new observation was how the intervention modified the contribution of leg strength to gait speed. Ankle plantarflexor strength increased 58.3% (p = 0.001) and its contribution to total leg strength still increased ∼4% (p<0.05). The intervention also increased hip extension strength 73.6% (p = 0.001) and its contribution to the total leg strength still increased ∼7% (p<0.05). In contrast, the knee extensor and hip abductor strength increased much less, 4 to 12%, and their contribution to the total leg strength did not change or even decreased ∼6% (knee extensors).

Our findings are especially insightful in combination with additional analysis of the data revealing that individual leg strength measures showed low or no association with gait speed at baseline (r = −0.01 to 0.55, 1 of 10 correlations p<0.05, Table 3). However, after the intervention stepwise regression analyses identified the hip extensors and ankle plantar flexors, muscle that increased most their contribution to the total leg strength, the only significant predictors of self-selected and maximal gait speed, respectively only in the training group (Figure 1). These findings agree with the predictions of biomechanical plasticity that assigns a putative role to hip and ankle function in the aging gait [23], [24].

Old compared with young adults tend to walk with reduced ankle plantarflexor output and it seems that an increase in hip mechanical output compensates for this reduction in an effort to maintain gait speed [23], [24]. Our data further specify this concept by showing a substantial increase in the correlation to r = 0.75 (p = 0.001) from r = 0.02 (p = 0.540) between ankle plantarflexor strength and maximal gait speed and to r = 0.58 (p = 0.020) from r = 0.06 (p = 0.840) between hip extensor strength and self-selected gait speed (Figure 1). The plantar flexors contribute over 70% to the total support power in the stance phase of gait in young adults which can decrease as much as 20% in old adults with 38% increase in hip power while walking at 1.0 to 1.5 m/s [24], [48]–[54]. The small or no increase after training in the correlation between hip abductor and adductor maximal strength and gait ability, respectively, underscores the specific role of plantarflexors and hip extensors play in gait speed (Table 2). The present data thus show that strength training may not have increased gait speed beyond control subjects’ speed but it produced a favorable reconfiguration of the individual joint contributions to gait velocity, providing possibly the first evidence for the incorporation of newly acquired strength into the mechanism that generates gait speed.

Leg press power training improved ankle plantarflexor and hip extensor MVCs but not knee extensor, hip abductor, and hip adductor MVC (all p>0.05). The 4% increase in knee extension MVC is unexpectedly low because preliminary testing in young adults revealed 92±22% activation of the vastus lateralis during leg press as a percent of maximal electromyogram measured during maximal isokinetic knee extension. Previous studies that used leg press training reported no knee extension data or used several additional exercises so that our knee extension data are not directly comparable with these studies [28], [29], [39], [40], [55]. Perhaps young adults used a different lower extremity alignment in the preliminary study than old adults in the intervention group, resulting in a different muscle activation pattern. Still, it seems that leg press power training at 30–40% of maximal load as a single exercise or embedded in multiple training exercises is safe and effective for the conditioning of multiple muscle groups in old adults who may also prefer these light vs. heavy loads recommended for high-intensity strength training [19], [41], [42], [56], [57]. Further, because fast compared with slow muscle contractions are associated with higher motor unit activation at the same load [58], power vs. high-force but low-velocity exercise training is expected to activate a greater number of motor units, resulting in larger neuromuscular and functionally more specific adaptations.

One limitation of the present study was a lack of comprehensive measures of activity of daily living. A second limitation was a lack of biomechanical assessments that could provide more detailed and mechanistic insights into how newly acquired physical abilities became incorporated into the mechanics of gait. Due to the small sample sizes it was not possible to determine if the reported findings differed between men and women.

## Conclusion

Light-load leg press power training improved leg press 1RM load 40.4% and 32.0% the maximal force of 5 leg muscles without improving gait speed more in the intervention vs. control groups. However, the intervention modified how 5 measures of leg strength contributed to gait speed: Hip extensors and ankle plantarflexors became the only significant predictors of self-selected and maximal gait speed, respectively. These data provide possibly the first evidence for the incorporation of newly acquired strength into the mechanism that generates gait speed.

## Supporting Information

Table S1 Measured variables of physical function test in both groups.
(XLSX)

Table S2 1RM and training load progression in training group.
(XLSX)
